# Detecting Volemic, Cardiac, and Autonomic Responses From Hypervolemia to Normovolemia *via* Non-Invasive ClearSight Hemodynamic Monitoring During Hemodialysis: An Observational Investigation

**DOI:** 10.3389/fphys.2022.775631

**Published:** 2022-04-28

**Authors:** Chih-Jun Lai, Chung-Chih Shih, Hsing-Hao Huang, Ming-Hung Chien, Ming-Shiou Wu, Ya-Jung Cheng

**Affiliations:** ^1^ Institute of Epidemiology and Preventive Medicine, National Taiwan University, Taipei, Taiwan; ^2^ Department of Anesthesiology, National Taiwan University Hospital, Taipei, Taiwan; ^3^ Department of Anesthesiology, National Taiwan University Cancer Center, Taipei, Taiwan; ^4^ Department of Internal Medicine, National Taiwan University Hospital, Taipei, Taiwan

**Keywords:** cardiac index, clearsight, end-stage renal disease, hemodialysis, hemodynamic monitoring

## Abstract

**Background:** Unstable hemodynamics are not uncommon during hemodialysis (HD), which involves a rapid volume depletion, taking the patient from hypervolemia toward euvolemia. Since uremic patients commonly have cardiovascular comorbidities, hemodynamic changes during HD may reflect interactions among the volemic, cardiac, and autonomic responses to gradual volume depletion during ultrafiltration. Accurate identification of inappropriate responses helps with precisely managing intradialytic hypotension. Recently, the non-invasive ClearSight was reported to be able to detect causes of intraoperative hypotension. In this prospective observational study, we aimed to determine whether ClearSight could be used to detect patterns in stroke volemic, cardiac, and vasoreactive responses during HD.

**Methods:** ClearSight was used to monitor chronic stable patients receiving maintenance HD. Data of mean arterial blood pressure (MAP), heart rate (HR), stroke volume index (SVI), cardiac index (CI), and calculated systemic vascular resistance index (SVRI) were obtained and analyzed to examine patterns in volemic, cardiac, and vasoreactive changes from T0 (before HD) until T8 in 30-min intervals (total 4 h).

**Results:** A total of 56 patients with a mean age of 60.5 years were recruited, of which 40 of them were men. The average ultrafiltration volume at T8 was 2.1 ± 0.8 L. The changes in MAP and HR from T0 to T8 were non-significant. SVI at T7 was significantly lower than that at T1, T2, and T3. CI at T4 to T8 was significantly lower than that at T0. SVRI was significantly higher at T3 to T8 than at T0. Pearson’s correlation coefficients between SVI and CI and between SVRI and MAP were positive at all time points. The correlation coefficients between SVRI and SVI and between CI and SVRI were significant and negative for all time points.

**Conclusion:** ClearSight was able to detect patterns in hypervolemia during HD and was well tolerated for 4 h. CI decreased significantly after T4, with slightly decreased SVI. Ultrafiltration volume was not correlated with changes in SVI or CI. The vascular tone increased significantly, and this counteracted the reduced cardiac output after T4. With simultaneous monitoring on SVI, CI, and SVRI during HD, therefore, hypotension could be detected and managed by reducing the filtration rate or administering inotrope or vasopressors.

**Trial Registration:** clinicaltrials.gov, ID: NCT03901794.

## Introduction

The goal of hemodialysis (HD) is to restore the intracellular and extracellular fluid environment through diffusion and ultrafiltration (Himmelfarb and Ikizler, 2010). Theoretically, HD is aimed at programmed volume removal, to take the patient from a hypervolemic state to either a euvolemic or a slightly hypovolemic state with well-maintained circulation. Although fluid removal (ultrafiltration) is believed to be the most important aspect of the process, hemodynamic management is also challenging in the event of unpredictable cardiac and vascular responses to fluid removal because of the high prevalence of cardiovascular disease in uremic patients undergoing HD (Cozzolino et al., 2018). Precise management of intradialytic hypotension is difficult without accurate identification of the cause *via* simultaneous monitoring of the volemic, cardiac, and vasoreactive responses to the gradual volume depletion during HD. Hypotension due to delayed vasoreactive responses to volume depletion can be managed by reducing the ultrafiltration rate, maintaining observation, and administrating vasoconstrictors. Hypotension due to decreased CI can be managed by stopping further volume depletion or administering inotropic to maintain adequate systemic circulation.

For patients receiving HD, it is preferable to use a non-invasive, well-tolerated, and continuous monitoring system to detect hemodynamic changes along with HD. In addition, the system should be able to demonstrate the trends with gradual volume depletion from hypervolemia, normovolemia, or even hypovolemic state. It may help on adjusting the goal and speed of ultrafiltration because different cardiac effects from chronic fluid overload was also reported in patients receiving HD (Antlanger et al., 2013). Most previous monitoring systems, such as stroke volume variation (SVV) (Yoshihara et al., 2017), Doppler echocardiography (Nitta et al., 1989), and photoplethysmography (PPG) signals (Sandberg et al., 2014), focused on volemic changes. However, there are many other associated risk factors for hypotension that are not investigated, such as shifts in extracellular volume, altered vasoregulation (Reilly, 2014), inappropriate reduction of the sympathetic tone, and an increase in venous capacity (Daugirdas, 2001). The low total peripheral resistance index (TPRI) and cardiac power index (CPI) have recently been reported to be associated with the highest 1-year mortality rates in patients receiving HD (Doenyas-Barak et al., 2019). Therefore, monitoring the autonomic and cardiac responses that accompany volemic changes is crucial for ensuring a well-maintained circulation, especially for high-risk patients. At present, since intradialytic hypotension is still considered the most important complication during HD (Radziszewski and Sulowicz, 2006), periodic blood pressure (BP) measurement with non-invasive blood pressure (NIBP) and heart rate (HR) monitoring remains the routine monitoring procedure during HD, although a continuous, non-invasive monitoring system has been reported to be preferable for each episode of HD for high-risk patients (Leypoldt and Lindsay, 1999). Few monitoring systems are able to determine volemic, cardiac, and vasoregulatory responses to HD simultaneously. Since parametric feedback is helpful for differentiating the volemic, cardiac, and vasoregulatory responses to HD, as well as for the optimal management of hemodynamic instability, a well-tolerated, non-invasive monitoring system that monitors multiple parameters is indicated.

ClearSight (Edwards Lifesciences, Irvine, CA, United States) is a validated non-invasive continuous BP monitoring system (Kim et al., 2019; Lee et al., 2020). It has been reported to reduce intraoperative hypotension (Maheshwari et al., 2018) by facilitating well-informed decisions about volume administration and the adjustment of the vascular tone in moderate-to-high-risk surgery (Martina et al., 2012.). In addition to continuously monitoring BP, ClearSight provides continuous measurements of the stroke volume index (SVI) and cardiac index (CI) as well as the calculated systemic vascular resistance index (SVRI) over time. In this observational study, we used both ClearSight and conventional NIBP monitor. We aimed to test whether the parameters SVI, CI, and calculated SVRI could reveal the trends in volemic, cardiac, and vasoconstrictive responses to gradual volume depletion during HD, which involves the unique situation of progressing from hypervolemia to normovolemia.

## Methods

We enrolled chronic stable patients receiving maintenance HD three times a week for at least 6 months at the outpatient HD unit of our hospital, National Taiwan University Hospital (a tertiary care center in Taipei, Taiwan), from June 2017 to November 2018. We recruited adult patients aged >20 years, who had sinus rhythm, and a stable dry weight but without evidence of fluid overload (i.e., they exhibited no clinical signs of fluid overload, such as shortness of breath). Patients were excluded if they had been admitted for thrombosis of vascular access or acute cardiovascular events (stroke, acute coronary syndrome, arrythmia including atrial fibrillation, or decompensating heart failure) in the previous 3 months or if they had Raynaud’s disease, peripheral arterial occlusion disease, or a local skin defect on hands or fingers. The Research Ethics Committee of our intuition approved (approval number: 201702064RIPB) the study, and all participants provided written informed consent prior to participating. Our investigation is registered at http://clinicaltrials.gov with the identifier NCT03901794.

### NIBP Monitoring Application

After arriving in the HD room, the patients lay on the bed, and the standard hemodynamic monitoring (including pulse oximetry and electrocardiography) was conducted. In addition, the ClearSight system was attached to the hand without an arteriovenous fistula. An appropriately sized finger cuff was applied to the middle phalanx of the second or third finger. Data were continuously collected throughout the HD session.

### High-Efficiency HD Protocol

High-efficiency HD was performed by the same group of staff in accordance with the standard protocol at our outpatient HD unit. The dialysis was delivered using the conventional HD machines (Fresenius 4,008, Fresenius Medical Care, Bad Homburg, Germany) using Fresenius dialyzers (FX60/FX80/FX100 and Polysulfone, Fresenius Medical Care). The blood flow was set between 200 and 350 ml/min, and the dialyzate flow was set to 500 or 800 ml/min. The default dialyzate composition was bicarbonate at 32 mEq/L, potassium at 2.0 mEq/L, sodium at 136–140 mEq/L, and calcium at 2.5–3.0 mEq/L. Each dialysis session lasted 4 h. All routine laboratory data were collected monthly.

The target volume for ultrafiltration was set before the initiation of HD. Intrahemodialytic hypotension (IDH) was recorded based on automatic NIBP by HD nurses. IDH was defined as pre-HD systolic blood pressure (SBP) minus minimum intradialytic SBP ≧30 mmHg and minimum intradialytic SBP<90 mmHg (Flythe et al., 2015).

### Data Collection and Analysis

We collected the following demographic data: age, sex, weight, height, clinical records (including diabetes mellitus, hypertension, and cardiac vascular disease), and number of years on dialysis. The following consecutive data, recorded at 30-min intervals, were prospectively collected from the patients’ records by HD nurses: ultrafiltration volume, sequential NIBP data, IDH, and the subsequent management. The data (mean arterial blood pressure [MAP], HR, SVI, and CI) were collected by another observer, from the initiation of HD (T0) to its conclusion 4 h later (T8) at 30-min intervals, from the continuous-monitoring data recorded by the ClearSight system. The ClearSight acquisition system allowed the export of raw data, including SBP to a spreadsheet (Microsoft Excel, Microsoft Corporation, Redmond, WA, United States ). The data for each patient used to calculate IDH were analyzed in MATLAB 2019b (MathWorks, Natick, MA, United States ). We presented the mean number of episodes per patient during hemodialysis. The SVRI was calculated by the ClearSight system based on the assumption that central venous pressure (CVP) taken as 10 cmH2O.

### Statistical Analyses

Continuous data are presented as mean and standard deviation. Repeated measures analysis of variance (ANOVA) with Bonferroni *post hoc* tests was used to statistically analyze the differences between the nine time points (T0 to T8, from 0 to 4 h in 30 min increments) in terms of ultrafiltration volume, MAP, CI, SVRI, SVI, and HR. To assess potential relationships between these variables at each time point, we used Pearson’s correlation coefficients. *p* values <0.05 were considered statistically significant. Analyses were performed using SPSS (SPSS Inc., Chicago, IL, United States).

## Results

### Patient Characteristics

We recruited *56* patients who were receiving chronic HD, of which 40 were male. They had a mean age of 60 years. Demographic data, comorbidities, and hemodialysis vintage are summarized in Table 1.

**TABLE 1:** Patient characteristics (N = 56).

Variable	
Age (years)	60.5 ± 14.1
Sex (male/female; n)	40/16
Height (cm)	164.9 ± 7.4
Weight (kg)	66.0 ± 15.7
BMI	24.09 ± 4.6
Comorbidities (n/%)	
Diabetes mellitus	19 (33.9%)
Hypertension	30 (53.6%)
Cardiac disease	17 (30.4%)
Dialysis vintage (months)	70.1 ± 55.7

Data presented are mean ± SD or n (%).

BMI, body mass index.

### Changes in Ultrafiltration Volume Changes

**FIGURE 1:**
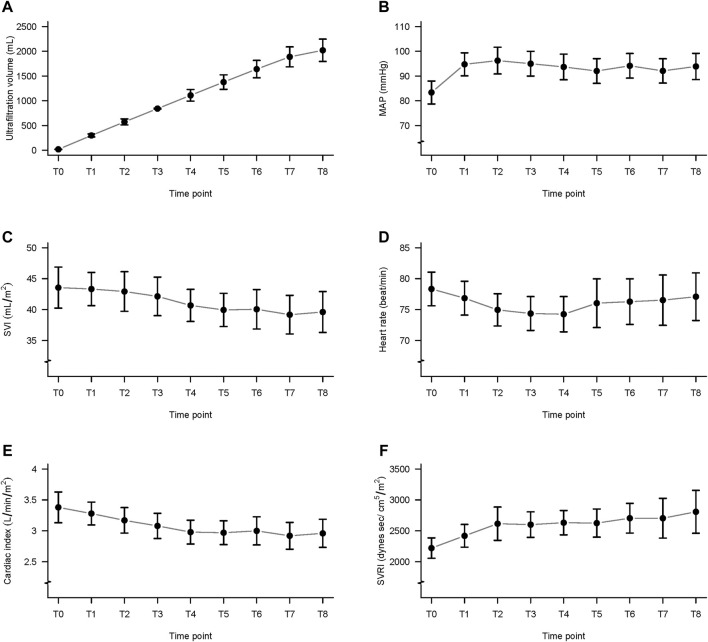
Ultrafiltration volume and data from the ClearSight monitoring system, during hemodialysis. **(A)** Ultrafiltration volume; **(B)** mean arterial pressure (MAP); **(C)** stroke volume index (SVI); **(D)** heart rate (HR); **(E)** cardiac index (CI); and **(F)** systemic vascular resistance index (SVRI); T0 to T8: from before hemodialysis (T0) to 240 min (T8) in 30-min intervals.

As shown in Figure 1A; Table 2, the ultrafiltration volume increased significantly from T0 to T8. The values for each time point were significantly different from those for all other time points (*p* < 0.001 for all comparisons). For example, the ultrafiltration volume value at T8 was significantly higher than that at T0. The average ultrafiltration volume at T8 was 2.1 ± 0.8 L, and the ultrafiltration rate was 8.2 ± 3.5 ml/kg/hr. The change in dry weight was −0.5 ± 0.18 kg. Hypotension was managed through close observation and reducing the ultrafiltration rate, which was sufficiently effective without medications. All patients completed their HD having achieved their preset goals.

**TABLE 2:** Trends in data on ultrafiltration volume and from the ClearSight monitoring system.

Variable	Time point									
	T0	T1	T2	T3	T4	T5	T6	T7	T8	*p*-value
Ultrafiltration volume (ml)	18.91 (7.65)	298.75 (115.50)	572.64 (224.37)	841.02 (33.44)	1109.48 (439.04)	1377.27 (547.66)	1639.55 (654.95)	1887.16 (752.15)	2019.32 (843.34)	<0.001
MAP (mmHg)	83.34 (17.32)	94.73 (17.38)	96.23 (20.17)	94.98 (18.65)	93.67 (19.35)	92.03 (18.71)	94.12 (18.57)	92.07 (18.37)	93.87 (19.78)	0.051
SVI (ml/m^2^)	43.56 (12.41)	43.33 (10.01)	42.93 (11.98)	42.14 (11.65)	40.68 (9.71)	39.95 (10.04)	40.06 (11.92)	39.18 (11.67)	39.61 (12.34)	0.002
HR (beat/min)	78.34 (10.14)	76.85 (10.23)	74.96 (9.74)	74.37 (10.23)	74.26 (10.64)	76.04 (14.71)	76.29 (13.77)	76.53 (15.19)	77.09 (14.38)	0.20
CI (L/min/m^2^)	3.38 (0.93)	3.28 (0.69)	3.17 (0.77)	3.08 (0.76)	2.98 (0.72)	2.97 (0.72)	3.00 (0.85)	2.92 (0.81)	2.96 (0.85)	<0.001
SVRI (dynes · s/cm^5^/m^2^)	2221.73 (614.26)	2419.54 (685.62)	2615.65 (1006.90)	2600.55 (772.51)	2632.18 (734.82)	2625.58 (850.68)	2704.68 (898.04)	2704.50 (1198.67)	2807.82 (1297.33)	0.005

Data are presented as mean (standard deviation); CI, cardiac index; HR, heart rate; MAP, mean arterial pressure; SVI, stroke volume index; SVRI, systemic vascular resistance index; T0 to T8, from before hemodialysis (T0) to 240 min later (T8) in 30-min intervals.

### Changes in MAP Detected by ClearSight

The MAP at T1 to T8 did not differ significantly from MAP before hemodialysis (T0) (Table 2; Figure 1B). The mean number of IDH episodes per patient was 16.74.

### Changes in SVI Detected by ClearSight

The SVI values differed significantly among the time points (Table 2; Figure 1C). The *post hoc* comparisons between the values at every time point are presented in Table 3. The values at T7 were significantly lower than those at T1, T2, and T3, and the differences were -4.15 ± 1.10 ml/m^2^ (*p* = 0.018), -3.75 ± 1.09 ml/m^2^ (*p =* 0.045), and -2.96 ± 0.85 ml/m^2^ (*p* = 0.041), respectively.

**TABLE 3:** Difference between time points in the stroke volume index.

Time point	T0	T1	T2	T3	T4	T5	T6	T7	T8
T0	0.00	−0.24 (1.13)	−0.63 (1.31)	-1.43 (1.44)	−2.88 (1.35)	−3.62 (1.59)	−3.51 (1.56)	−4.38 (1.46)	−3.96 (1.64)
T1		0.00	−0.40 (0.85)	−1.19 (1.07)	−2.64 (0.98)	−3.38 (1.21)	−3.28 (1.23)	−4.15 (1.10) ^*^	−3.73 (1.49)
T2			0.00	−0.79 (0.84)	−2.25 (1.07)	−2.98 (1.37)	−2.88 (1.15)	−3.75 (1.09) ^*^	−3.33 (1.46)
T3				0.00	−1.45 (0.94)	−2.18 (1.27)	−2.09 (0.98)	−2.96 (0.85) ^*^	−2.54 (1.27)
T4					0.00	−0.74 (0.61)	−0.63 (0.86)	−1.50 (0.73)	−1.08 (1.01)
T5						0.00	0.10 (1.01)	−0.77 (0.86)	−0.35 (1.06)
T6							0.00	−0.87 (0.71)	−0.45 (1.22)
T7								0.00	0.42 (0.97)
T8									0.00

Difference, the value in row 1 minus the value in column 1. T0 to T8, from before hemodialysis (T0) to 240 min later (T8) in 30-min intervals. ^*^:*p* < 0.05.

### Changes in HR Detected by ClearSight

The HR at T1 to T8 did not differ significantly from T0 (Table 2; Figure 1D).

### Changes in CI Detected by ClearSight

The CI values differed significantly among the time points (Table 2; Figure 1E). The *post hoc* comparisons of values at every time point are shown in Table 4. The CI at T4, T5, T6, T7, and T8 was significantly lower than that at T0, and the differences were -0.39 ± 0.10 (*p* = 0.01), -0.41 ± 0.10 (*p* = 0.01), -0.38 ± 0.11 (*p* = 0.044), -0.46 ± 0.10 (*p* = 0.001), and -0.42 ± 0.12 (*p* = 0.03), respectively.

**TABLE 4:** Difference between time points in the cardiac index.

Time point	T0	T1	T2	T3	T4	T5	T6	T7	T8
T0	0.00	−0.09 (0.86)	−0.21 (0.09)	−0.29 (0.10)	−0.39 (0.09) ^**^	−0.41 (0.10) ^**^	−0.38 (0.11) ^*^	−0.47 (0.10) ^***^	−0.42 (0.17)^*^
T1		0.00	−0.12 (0.06)	−0.21 (0.08)	−0.30 (0.08) ^**^	−0.32 (0.08) ^*^	−0.28 (0.09)	−0.37 (0.09)^**^	−0.32 (0.11)
T2			0.00	−0.08 (0.06)	−0.18 (0.08)	−0.19 (0.09)	−0.16 (0.09)	−0.25 (0.08)	−0.20 (0.17)
T3				0.00	−0.09 (0.08)	−0.11 (0.09)	−0.08 (0.07)	−0.16 (0.07)	−0.12 (0.09)
T4					0.00	−0.01 (0.06)	0.02 (0.06)	−0.06 (0.06)	−0.02 (0.08)
T5						0.00	0.03 (0.06)	−0.05 (0.06)	−0.01 (0.08)
T6							0.00	−0.08 (0.05)	−0.04 (0.08)
T7								0.00	0.04 (0.08)
T8									0.00

Difference, the value in row 1 minus the value in column 1. T0 to T8, from before hemodialysis (T0) to 240 min later (T8) in 30-min intervals. ^*^: *p* < 0.05; ^**^: *p* < 0.01;^***^: *p* < 0.001.

### Changes in SVRI Detected by ClearSight

The calculated SVRI was significantly higher at T3 to T8 than that at T0 (Table 2; Figure 1F). The *post hoc* comparisons of the values at every time point are shown in Table 5. The differences were 378.82 ± 90.21 (*p* = 0.005), 410.46 ± 81.96 (*p* < 0.001), 408.86 ± 107.28 (*p* = 0.02), 482.96 ± 111.73 (*p* = 0.003), 532.78 ± 146.89 (*p* = 0.03), and 586.09 ± 160.60 (*p* = 0.03).

**TABLE 5:** Difference between time points in the systemic vascular resistance index.

Time point	T0	T1	T2	T3	T4	T5	T6	T7	T8
T0	0.00	197.81 (67.9)	393.92 (118.43)	378.82 (90.21) ^**^	410.46 (81.96) ^***^	408.86 (107.28) ^*^	482.96 (111.73) ^**^	532.78 (146.89) ^*^	586.09 (160.60)^*^
T1		0.00	196.11 (110.38)	181.02 (94.77)	212.65 (86.46)	206.05 (119.76)	285.15 (120.81)	334.96 (161.33)	388.28 (178.21)
T2			0.00	−15.09 (112.56)	16.54 (125.21)	9.94 (148.21)	89.04 (136.47)	138.86 (180.48)	192.17 (189.55)
T3				0.00	31.63 (65.91)	25.04 (94.36)	104.14 (77.36)	153.95 (133.10)	207.27 (140.13)
T4					0.00	-6.59 (72.65)	72.51 (68.90)	122.32 (126.60)	175.64 (135.45)
T5						0.00	79.10 (63.85)	128.92 (132.71)	182.24 (157.86)
T6							0.00	49.81 (121.11)	103.13 (148.87)
T7								0.00	53.32 (99.11)
T8									0.00

Difference, the value in row 1 minus the value in column 1. T0 to T8, from before hemodialysis (T0) to 240 min later (T8) in 30-min intervals. ^*^: *p* < 0.05; ^**^: *p* < 0.01; ^***^: *p* < 0.001.

### Pearson’s Correlations Between Time Points

The relationships between CI, SVI, SVRI, HR, MAP, and ultrafiltration volume at all time points are presented in Table 6. The range of Pearson’s correlation coefficient between CI and SVI was 0.76–0.87 among all time points (T0 to T8, *p* < 0.001 for all). The range of Pearson’s correlation coefficients between SVRI and SVI was −0.55 to −0.70 among all time points (*p* < 0.001 for all). The range of Pearson’s correlation coefficients between SVRI and CI was −0.72 to −0.76 among all time points (*p* < 0.001 for all). The range of Pearson’s correlation coefficient between SVRI and MAP was 0.41 (T0, *p* = 0.002) and 0.55 to 0.68 among all the time points except T0 (*p* < 0.001 for all except T0).

**TABLE 6:** Pearson’s correlation coefficients between parameters for time points.

Before hemodialysis (T0)	SVI	CI	MAP	HR	SVRI
Ultrafiltration volume	0.10	0.17	0.03	0.11	−0.14
SVI		0.87***	0.17	−0.26	−0.70***
CI			0.17	0.22	−0.76***
MAP				0.40	0.41**
HR					−0.09
**After 30-min hemodialysis (T1)**					
Ultrafiltration volume	0.04	0.11	0.24	0.10	0.07
SVI		0.81***	−0.22	−0.36**	−0.70***
CI			−0.11	0.23	−0.78***
MAP				0.15	0.65***
HR					0.10
**After 60-min hemodialysis (T2)**					
Ultrafiltration volume	−0.14	0.01	0.10	0.23	0.07
SVI		0.84***	−0.13	−0.35**	−0.61***
CI			−0.09	0.19	−0.73***
MAP				0.02	0.68***
HR					-0.17
**After 90-min hemodialysis (T3)**					
Ultrafiltration volume	−0.31*	0.03	−0.16	−0.03	0.06
SVI		0.76***	0.42	0.23	−0.56***
CI			0.002	0.29*	−0.73***
MAP				0.53***	0.64***
HR					−0.23
**After 120-min hemodialysis (T4)**					
Ultrafiltration volume	−0.10	0.05	0.10	0.20	0.07
SVI		0.79***	0.06	−0.30*	−0.61***
CI			0.13	0.33*	−0.72***
MAP				0.10	0.55***
HR					−0.20
**After 150-min hemodialysis (T5)**					
Ultrafiltration volume	−0.16	−0.04	0.07	0.08	0.14
SVI		0.78***	0.01	−0.34*	−0.59***
CI			−0.02	0.30*	−0.76***
MAP				−0.12	0.58***
HR					−0.31*
**After 180-min hemodialysis (T6)**					
Ultrafiltration volume	−0.05	0.05	0.02	0.13	−0.05
SVI		0.80***	−0.11	−0.29*	−0.65***
CI			−0.10	0.32*	−0.80***
MAP				−0.02	0.58***
HR					-−0.27*
**After 210-min hemodialysis (T7)**					
Ultrafiltration volume	−0.07	0.08	−0.03	0.20	−0.03
SVI		0.75***	−0.04	−0.45***	−0.55***
CI			−0.11	0.23	−0.70***
MAP				−0.12	0.65***
HR					−0.14

**After 240-min hemodialysis (T8)**					
Ultrafiltration volume	−0.12	0.02	−0.02	0.22	0.01
SVI		0.82***	−0.03	−0.45***	−0.57***
CI			−0.23	0.13	−0.75***
MAP				−0.26	0.67***
HR					−0.14

Data are presented as mean (standard deviation); CI, cardiac index; HR, heart rate; MAP, mean arterial pressure; SVI, stroke volume index; SVRI, systemic vascular resistance index; T1 to T8, from before hemodialysis (T0) to 240 min later (T8) in 30-min intervals. ^*^: *p* < 0.05; ^**^: *p* < 0.01; ^***^: *p* < 0.001.

## Discussion

Our results show that ClearSight is able to detect patterns in volemic, cardiac, and vasoreactive changes even in uremic patients starting from the hypervolemic state. ClearSight is a well-tolerated, non-invasive monitoring system for 4-h HD and is not affected by the patient moving their hands or eating during the HD session. We detected 16.74 episodes per patient with gradual volume depletion during HD, although our results revealed similar blood pressure throughout HD. We detected 16.74 episodes per patient of hypotension, and based on simultaneous and continuous monitoring, this was not associated with low SVI or CI. Close observation and reducing the HD rate were effective for returning BP to normal levels without medications. Based on our results, the most intradialytic hypotension was associated with delayed or inadequate vasoreactivity during each 30-min interval but not with ultrafiltration volume. Reducing the filtration rate may be effective for stabilizing BP long enough for adequate autonomic responses to be initiated. Once the BP had normalized, the filtration rate was returned to its original value, and HD was completed.

The direction of the changes in the volume status during HD reflected the patients’ progression from a hypervolemic plateau back to the normally responsive range, according to Flank–Starling law (Malbrain et al., 2015). The SVI decreased slightly but remained relatively constant from T0 to T6. Based on our results, the decreases in SVI were not correlated with ultrafiltration volume. The patterns of change in SVI, when the patient progresses from hypervolemia to euvolemia, are rarely discussed in the literature, relative to those in hypovolemia. In the hypervolemic state (T0 to T2), SVI remained relatively constant in spite of the higher rate of fluid removal with a relatively constant SVRI. The plasma refill may occur *via* refilling of the vascular space from the interstitial and cellular compartments or from venous blood reservoir (Greenway and Lister, 1974). The SVI would therefore be well-maintained despite the change in volume because of the increased venous capacity that exists in uremic patients before HD.

Our results revealed an inflection point at which the vasoreactive response is activated. SVRI did not increase significantly until T3, but thereafter (T3 to T8) it increased and was correlated with the ultrafiltration volume. The passive leg raising (PLR) test has been shown to effectively identify vasopressor-dependent circulatory shock (Krige et al., 2016). Another study, in which PLR was performed during the second hour of HD, found that BP increased during PLR (Erdem, 2016). Our results agree with that report; however, if PLR was performed before HD, it was impossible to identify a hypervolemic state based on it.

The SVRI acts as a sentinel for autonomic responses to volume depletion under normovolemic or hypovolemic conditions that serve to maintain adequate circulation. A consistently elevated SVRI also indicates well-compensated vasoconstriction. When the patterns of change in SVRI and CI are similar, it is possible to differentiate autonomic and cardiac responses to gradual volume depletion. Simultaneous monitoring may help to manage unstable hemodynamics because possible cardiac stunning associated with HD has been previously reported (Power et al., 2016). However, according to the same assumption about CVP that we used to calculate SVRI in this study, the slope of the change in SVRI should be much steeper in that situation than it is in the illustrated figure.

We recorded 16.74 IDH episodes per patient during HD. The changes in CI are crucial for identifying inadequate circulation and determining necessary management steps, such as initiating treatment with inotropic drugs or vasopressors. Our results showed that CI decreased significantly from T4 onward but was nevertheless well maintained until T8. The CI data obtained in this study are similar to those obtained using another non-invasive bioreactance cardiac output monitoring system (NICOM) (Bernardo et al., 2015). Since we did not include patients with cardiac dysfunction, CI remained within the normal range throughout HD. However, the clinical importance of monitoring CI may differ between uremic patients with and without cardiovascular diseases (Fuehrlein et al., 2007). With the continuous monitoring of SVI, any further decrease in SVI or CI can be detected before severe hypotension occurs. Our results showed that the hypotension that occurred during HD was associated with temporally inappropriate vasoreactivity but not low stroke volume or CI.

The monitoring trends in SVI, HR, CI, and SVRI should be beneficial during HD for high-risk patients with limited cardiac function, possible autonomic neuropathy, unknown pre-HD volume status, comorbidity manifestations, or restricted protein or fluid intake (Chan et al., 2019). In addition to identification of hypotension, it would facilitate the identification of pre-HD hypovolemia, adjustment of filtration goals, or provision of early cardiac support, which may prevent cardiovascular problems associated with HD. However, ClearSight may be too costly for routine application during each HD session, and specific expertise is required for differential diagnosis. Based on the principles of “goal-directed dialysis care” (Chan et al., 2019), we suggest that ClearSight is used for patients with higher cardiovascular risks before their chronic stability is established.

The study has some limitations as follows: first, the effect of patients’ medication on any associated underlying disease could not be evaluated. Second, our study had a small sample size. Third, we assumed a CVP level of 10 cmH_2_O, which may have been a limitation in terms of obtaining a reliable continuous estimation of SVRI. Fourth, the more detailed hemodynamic changes in the parameters measured using ClearSight particularly CI and the relevant mechanisms associated with IDH should be further investigated, even though we did assess IDH in this study.

In conclusion, ClearSight is a well-tolerated practical system for simultaneous monitoring of the volume status and the autonomic response, even under gradual volume depletion from a hypervolemic status. By observing the trends in systemic vascular resistance and cardiac output in addition to blood pressure, this easy-to-use, non-invasive monitoring system may provide more information on determining pre-HD status, responses to ultrafiltration, and effective hemodynamic managements during HD.

## Data Availability

The original contributions presented in the study are included in the article/Supplementary Materials, further inquiries can be directed to the corresponding author.
